# Kefir Grains Change Fatty Acid Profile of Milk during Fermentation and Storage

**DOI:** 10.1371/journal.pone.0139910

**Published:** 2015-10-07

**Authors:** C. P. Vieira, T. S. Álvares, L. S. Gomes, A. G. Torres, V. M. F. Paschoalin, C. A. Conte-Junior

**Affiliations:** 1 Food Science Program, Universidade Federal do Rio de Janeiro, Rio de Janeiro, Rio de Janeiro, Brazil; 2 Nutrition Institute, Universidade Federal do Rio de Janeiro, Macaé, Rio de Janeiro, Brazil; 3 Department of Food Technology, Universidade Federal Fluminense, Niterói, Rio de Janeiro, Brazil; Agricultural University of Athens, GREECE

## Abstract

Several studies have reported that lactic acid bacteria may increase the production of free fatty acids by lipolysis of milk fat, though no studies have been found in the literature showing the effect of kefir grains on the composition of fatty acids in milk. In this study the influence of kefir grains from different origins [Rio de Janeiro (AR), Viçosa (AV) e Lavras (AD)], different time of storage, and different fat content on the fatty acid content of cow milk after fermentation was investigated. Fatty acid composition was determined by gas chromatography. Values were considered significantly different when *p*<0.05. The highest palmitic acid content, which is antimutagenic compost, was seen in AV grain (36.6g/100g fatty acids), which may have contributed to increasing the antimutagenic potential in fermented milk. Higher monounsaturated fatty acid (25.8g/100g fatty acids) and lower saturated fatty acid (72.7g/100g fatty acids) contents were observed in AV, when compared to other grains, due to higher Δ9-desaturase activity (0.31) that improves the nutritional quality of lipids. Higher oleic acid (25.0g/100g fatty acids) and monounsaturated fatty acid (28.2g/100g fatty acids) and lower saturated fatty acid (67.2g/100g fatty acids) contents were found in stored kefir relatively to fermented kefir leading to possible increase of antimutagenic and anticarcinogenic potential and improvement of nutritional quality of lipids in storage milk. Only high-lipidic matrix displayed increase polyunsaturated fatty acids after fermentation. These findings open up new areas of study related to optimizing desaturase activity during fermentation in order to obtaining a fermented product with higher nutritional lipid quality.

## Introduction

Kefir grains are a starter culture used for producing kefir beverage, obtained by fermenting milk with kefir grains. These grains contain a complex symbiotic community of lactic acid bacteria (LAB) (*Lactobacillus*, *Lactococcus*, *Streptococcus*, *Enterococcus*, *Leuconostoc*), yeasts (*Kluyveromyces*, *Saccharomyces* and *Torula*) and acetic acid bacteria (*Acetobacter*) confined in a matrix of polysaccharides and proteins [1]. Furthermore, probiotic bacteria in kefir grains have shown many health-promoting properties, such as inhibition of detrimental effect of pathogens, enhancement of the intestinal barrier and modulation of the immune response. These health benefits have been observed both *in vitro* and *in vivo* experiments [2, 3].

Fatty acids such as butyric acid (4:0), oleic acid (18:1n9), palmitic acid (16:0) and conjugated linoleic acid, which have shown antimutagenic and/or anticarcinogenic properties, may be altered in their proportions by fermentation [4–7]. Yogurt has shown antimutagenic effects against a range of mutagens and promutagens, in microbial and mammalian cell systems [8]. Epidemiological studies have shown that consumption of fermented dairy products can reduce the risk of colorectal cancer [9, 10].

Nutritional quality indexes of lipids compare and quantify saturated fatty acids (SFA) and certain unsaturated fatty acids, and these are important nutritional indexes since SFA are considered promoters of thrombus formation [11]. On the other hand, polyunsaturated fatty acids (PUFA) and monounsaturated fatty acids (MUFA) reduce the formation of atherosclerotic plaques and decrease the level of esterified fatty acids, cholesterol, and phospholipids [12]. Thus, such indexes can be used to indicate an increased or reduced risk for cardiovascular diseases as well as potentially healthy food can be considered [13]. Furthermore, it is known that lactic acid bacteria can have increased desaturase activity (DA) during stress conditions such as fermentation [14]. Desaturase activity is used in order to estimate the conversion ratio of saturated fatty acids in unsaturated fatty acids [15]; therefore, an increase of desaturase activity of microorganisms present in food is desirable.

Studies have reported that the fermentation by lactic acid bacteria (LAB) of dairy products is able to affect the composition of fatty acids, leading to an increase or decrease of analyzed fatty acids [16, 17]. Furthermore, it seems that fat content in the food matrix can induce changes in fatty acid profile. Remagni et al. [18] observed changes in palmitic (16:0), stearic (18:0) and total saturated and unsaturated acids when cholesterol was added to the growth media. Serafeimidou et al. [19] compared the composition of fatty acid in yogurt produced from either skim milk or whole milk, encountering the highest amounts of saturated fatty acids, palmitic acid (16:0) and lauric acid (12:0) in yogurts made from skim milk. Because lactic acid bacteria for the food industry often undergo long-term storage, the effect of storage time on fatty acids profile in food matrix has been studied [20]. Gursoy et al. [21] observed that different bacterial cultures (*Enterococcus*, *Lactobacillus* and *Bifidobacterium*) had a significant effect on conjugated linoleic acid concentration and fatty acid composition of white pickle cheese.

Several studies have reported that lactic acid bacteria may increase the production of free fatty acids by lipolysis of milk fat [22, 23]. It can lead to change in free fatty acids composition, including linoleic acid (18:2n6), which is substrate for CLA production [24]. However, studies showing the effect of kefir grains on fatty acids composition as well as storage time and dairy matrix influence on its composition were not found.

The aim of the present study was to investigate the influence of kefir grain origins, storage time and types of dairy matrix on fatty acid profile, including CLA isomers, in milk after fermentation. The chemical characteristics of kefir beverage were also evaluated.

## Materials and Methods

### Reagents

All organic solvents used were of chromatographic grade (Sigma). A commercial mixture of fatty acid methyl esters (FAME mix; Supelco, Co., ref.47885-U) was used for identification of FAME peaks. All the other reagents were of chromatographic grade (Sigma Chemical Co., USA).

### Geographical origin of samples

The three different kefir grains used in this study were collected from different cities in southeastern Brazil (AR, Niterói, Rio de Janeiro; AV, Viçosa, Minas Gerais; and AD, Lavras, Minas Gerais). Grains AV and AD were kindly provided by researchers from Brazilian universities (Universidade Federal de Lavras-UFLA and Universidade Federal de Viçosa-UFV), whereas the third grain (AR) belonged to a family that traditionally cultivated the kefir grain in private household for self-consumption. The complete description of the microbial communities of these kefir grains was previously performed by PCR-DGGE and pyrosequencing analysis [25]. No permits are required for the described study, which is based entirely on kefir grains that were previously studied by our research group [25, 26]. Thus, other previously published data about these grains are accessible to the public; this research complies with all relevant regulations.

### Fermentation conditions of kefir grains

Kefir grains (AD, AV and AR) were activated (10% w/v) in sterile reconstituted semi-skim milk (1.45% fat) at 25°C for 24 h, filtered to remove the clotted milk, and rinsed with sterile water. The activation step was repeated three times [27]. The activated grains (6 g) were inoculated in commercial UHT semi-skim milk (500 mL) and statically incubated at 25°C for 24 h. Samples (1.5 mL) of the beverage were aseptically taken. Triplicate fermentations were performed.

AR grains showed, in general, a higher detrimental effect on fatty acids profile and on nutritional quality index/ratio of lipids when compared to other kefir grains. Thus, in order to analyze if time of storage is capable of improving these parameters of nutritional quality, only AR grains activated and incubated according to the conditions outlined above were stocked to 4°C for 14 days after fermentation.

The influence of dairy matrix on fatty acid profile was studied through inoculum AD grains both commercial UHT whole and semi-skim milk according to fermentation conditions described above (25°C for 24 h).

### Chemical characteristics

Protein percentage was analyzed according to Kjeldahl Method, base on the protein nitrogen content of the kefir samples and then expressed as protein percentage by multiplying the nitrogen content determined by 6.38 [28]. Titratable acidity was determined according to AOAC [28]. The digital pH-meter (ISTEK, Model 720P. Korea) was calibrated with standard buffer solutions pH 4.0 and 7.0 and used for measuring the pH kefir samples [29]. Total fat from kefir samples extracted from freeze-dried samples with light petroleum ether by Soxhlet method and determined gravimetrically [28].

### Analysis and derivatization of fatty acids

Samples of kefir (1.5 mL) were dispersed in 3.6 mL isopropanol with a high-throughput homogenizer (Ultra-turrax T18 basic model; IKA^®^-Works do Brasil Ltda.; Rio de Janeiro, Brazil) for 30s; afterwards, the volumes of *n*-hexane were adjusted for a final proportion of hexane:isopropanol of 3:2 (v/v), followed by dispersion for more 60s. The lipid extracts were then filtered through sinterized glass with medium porosity [30]. The residues were washed with 2.5mL of hexane:isopropanol (3:2, v/v). The solvents were removed from the filtrates with a gentle N_2_ stream, in which the lipid extracts were suspended in 10 mL of dicloromethane:methanol (10:1, v/v) and stored at -20°C until derivatization.

The transesterification of fatty acids was performed as describe by Kramer et al. [31]. In brief, lipid extracts containing 15 mg of lipids were dried with a gentle N_2_ stream and dissolved in 300 μL of NaOCH_3_ solution in methanol (0.3 mol/L). Samples were heated under N_2_ in a water bath (50°C for 10 min) with agitation. After cooling, 100 μL of HCl (10%, w/v) in methanol was added and heated in a water bath (80°C for 10 min) with agitation. The fatty acid methyl esters (FAMEs) extraction was performed with 500 μL of hexane by centrifugation, after addition of 1.2 mL aqueous NaCl solution (28%, w/v). The superior hexane layer was evaporated with a gentle N_2_ stream and the FAME were suspended in 1.2 mL of hexane and stored (-20°C) until analysis by gas chromatography (GC).

### Analysis of FAME by gas chromatography

The resultant FAMEs were analyzed by gas chromatography using GC–2010 gas chromatograph (Shimadzu, Japan) equipped with a flame ionization (FID) and a split injection system (ratio of 1:30) and fitted with a capillary column (30m x 0.32mm inner diameter, 0.25μm film; Omegawax–320, Supelco Co., EUA). The injector and detector were operated at 260° and 280°C, respectively. The oven temperature was held at 40°C for 3min, temperature programmed at 2.5°C/min to 180°C, then temperature was programmed at 2.0°C/min to 210°C and then held for 25min. Helium was used as a carrier gas and column pressure was set to attain a carrier gas speed of 25.0cm/s.

Gas-chromatographic peaks of FAME of the samples were identified by comparison of the retention time data with that of standards. Heptadecanoic acid (C_17:0;_ Sigma Chemical Co.) was used as an internal standard for quantification.

### Nutritional quality indexes of lipids

Proatherogenic and Antiatherogenic fatty acids are related by the index of atherogenicity (AI) while prothrombogenic and antithrombogenic fatty acids are related by the index of thrombogenicity (TI). Thus, the lower these indexes are, the more potentially healthy food is considered [13]. Hypocholesterolemic fatty acids/hipercholesterolemic fatty acids (HH) index is related more specifically to cholesterol metabolism, which is the ratio between hypocholesterolemic fatty acids and hypercholesterolemic fatty acids. Thus, greater values of HH indicate better nutritional quality [32]. Another indicator of nutritional quality is the PUFA/SFA ratio which is recommended to be above 0.4 by health guidelines in order to prevent an excess of saturated fatty acids with a detrimental effect on the LDL cholesterol plasmatic level [33].

The nutritional quality of the food lipid fraction was analyzed through five indexes from fatty acids composition data which were expressed as percent of the total identified fatty acids (Tables 1, 2 and 3). The indexes of atherogenicity and thrombogenicity were calculated as proposed by Ulbricht and Southgate [34] and fatty acids hypocholesterolemic/hypercholesterolemic ratio calculated according Santos-Silva et al. [35] as follows:

AI=[(C12:0)+(4x C14:0)+(C16:0)]/(∑MUFA+∑n6+∑n3)

TI=[(C14:0+C16:0+C18:0)]/[(0.5x∑MUFA)+(0.5x∑n6)+(3x∑n3)+(∑n3/∑n6)]

HH=(C18:1cis9+C18:2n6+C20:4n6+C18:3n3+C20:5n3+C22:5n3+C22:6n3)/(C14:0+C16:0)

**Table 1:** Chemical characteristics of semi-skim milk and milk fermented with kefir grains of different origins (AD, AV and AR) for 24h.

Chemical characteristics	Semi-skim milk		AV		AR		AD	
	Mean	SD	Mean	SD	Mean	SD	Mean	SD
pH***	6.69^a^	0.01	4.00^b^	0.05	4.08 ^b^	0.01	4.05 ^b^	0.05
Acidity (°D)***	16.33^a^	0.58	104.31^b^	1.68	102.41 ^b^	1.68	105.22 ^b^	1.70
Protein (%)**	1.03^a^	0.00	5.94 ^b^	0.11	5.98 ^b^	0.23	5.94 ^b^	0.04
Fat (%)	1.45	0.01	1.50	0.05	1.52	0.03	1.48	0.08

SD: standard deviation ^a,b^Means within the same row with different superscripts are significantly different. ANOVA (p<0.05) with Tukey´s post-hoc test

**p<0.01

***p<0.001.

**Table 2:** Fatty acids composition (g/100g total fatty acids) in semi-skim milk and semi-skim milk fermented with kefir grains from different origins (AD, AV and AR) for 24h.

Fatty acids	Semi-skim milk		AV		AR		AD	
	Mean	SD	Mean	SD	Mean	SD	Mean	SD
6:0	1.17	0.26	1.54	1.34	0.59	0.39	ND	-
8:0	1.44	0.43	ND	-	0.85	0.42	ND	-
10:0	5.01	0.06	2.75	0.13	3.41	1.19	1.28	1.52
12:0*	2.65^b^	0.22	3.24^a.b^	0.16	7.45^a^	3.27	2.06^b^	2.10
13:0	ND	-	ND	-	0.38	0.21	0.24	0.05
14:0*	10.8^a.b^	0.68	12.9^a^	0.29	8.13^b^	2.55	8.84^a.b^	6.28
14:1n5	2.21	0.37	ND	-	2.10	0.42	1.77	1.23
15:0***	1.29^b^	0.08	1.42^b^	0.03	24.4^a^	11.47	0.67^b^	0.00
15:1n5	0.31	0.01	ND	-	2.35	1.06	0.20	0.20
16:0*	29.9^b^	0.87	36.6^a^	1.34	29.4^b.c^	0.06	24.5^c^	0.22
16:1n7	2.10	0.12	1.11	0.97	1.84	0.57	2.30	0.58
17:1n7	0.25	0.23	ND	-	0.29	0.18	ND	-
18:0***	13.6^b^	0.26	14.7^b^	1.12	17.2^b^	2.30	39.7^a^	2.77
18:1n9***	25.9^a^	0.81	24.7^a^	1.97	2.37^b^	0.17	3.98^b^	0.03
18:2n6t	ND	-	ND	-	0.15	0.07	0.15	0.14
18:2n6c	2.38	0.20	2.25	0.03	1.07	0.88	2.92	0.39
18:3n6	ND	-	ND	-	0.25	0.02	0.57	0.07
18:3n3	0.40	0.04	ND	-	0.22	0.11	0.28	0.05
18:2;9c11t	1.25	0.12	ND	-	0.82	0.16	1.12	0.18
18:2;10t12c	0.44	0.13	ND	-	ND	-	0.19	0.17
18:2;10c12c	0.31	0.04	ND	-	ND	-	0.23	0.05
18:2;tt	0.37	0.05	ND	-	ND	-	ND	-
20:0	ND	-	ND	-	0.12	0.02	0.26	0.06
20:1n9	ND	-	ND	-	ND	-	0.13	0.12
20:2n6	ND	-	ND	-	0.32	0.01	0.50	0.28
20:3n6	ND	-	ND	-	0.14	0.13	0.32	0.07
20:4n6	ND	-	ND	-	ND	-	ND	-
20:3n3	ND	-	ND	-	ND	-	ND	-
20:5n3	ND	-	ND	-	ND	-	ND	-
21:0	ND	-	ND	-	ND	-	ND	-
22:0	ND	-	ND	-	0.35	0.37	ND	-
22:1n9	ND	-	ND	-	ND	-	0.18	0.16
22:2n6	ND	-	ND	-	0.28	0.48	1.70	0.45
22:6n3	ND	-	ND	-	ND	-	ND	-
23:0	ND	-	ND	-	ND	-	ND	-
24:0**	ND	-	ND	-	6.04^b^	0.89	15.6^a^	1.80
24:1n9	ND	-	ND	-	ND	-	ND	-
**Sums of fatty acids**								
SFA***	64.1^c^	0.69	72.7^b^	0.34	88.6^a^	1.51	84.8^a^	0.37
MUFA*	30.8^a^	0.73	25.8^b^	1.00	8.16^c^	0.81	7.23^c^	0.60
PUFA*	5.14^a.b^	0.17	1.50^b^	1.30	3.27^b^	1.10	7.99^a^	0.80
n–3*	0.39^a^	0.04	0.00^d^	0.00	0.15^c^	0.01	0.28^b^	0.05
n–6*	3.56^b^	0.11	2.25^b^	0.03	2.23^b^	1.05	6.76^a^	1.21
**Ratio and indices**								
PUFA/SFA**	0.08^a^	0.00	0.02^b^	0.02	0.04^b^	0.01	0.09^a^	0.01
AI***	2.18^b^	0.07	3.25^b^	0.07	6.53^a^	0.75	3.33^b^	0.16
TI***	2.94^b^	0.07	4.57^b^	0.02	9.35^a^	1.38	9.36^a^	0.81
HH*	0.74^a^	0.02	0.53^ab^	0.01	0.09^c^	0.03	0.25^bc^	0.01
DA***	0.39^a^	0.00	0.31^b^	0.00	0.12^c^	0.00	0.15^c^	0.00

ND: not detected; SD: standard deviation; SFA- saturated fatty acid; MUFA- monounsaturated fatty acid; PUFA- polyunsaturated fatty acid; AI- index of atherogenicity; TI- index of thrombogenicity; DA- desaturase activity; HH- hypocholesterolemic fatty acids/hipercholesterolemic fatty acids ^a,b,c^Means within the same row with different superscripts are significantly different. ANOVA (p<0.05) with Tukey´s post-hoc test *p<0.05

**p<0.01

***p<0.001.

**Table 3:** Evolution of fatty acids (g/100g total fatty acids) during 24h of fermentation and 14 days of storage in semi-skim milk with AR kefir grain.

Variable	Semi-skim milk		24h		14d	
	Mean	SD	Mean	SD	Mean	SD
**Individual fatty acids**						
6:0	1.17	0.26	0.59	0.39	0.35	0.31
8:0	1.44	0.43	0.85	0.42	0.43	0.39
10:0	5.01	0.06	3.41	1.19	1.47	0.59
12:0**	2.65^a.b^	0.22	7.45^a^	3.27	1.88^b^	0.58
14:0	10.8	0.68	8.1	2.55	7.8	1.72
14:1n5	2.21	0.37	2.10	0.42	1.31	0.39
15:0***	1.29^b^	0.08	24.4^a^	11.47	3.10^b^	1.09
15:1n5	0.31	0.01	2.35	1.06	0.16	0.14
16:0	29.9	0.87	29.4	0.06	25.5	1.90
16:1n7	2.10	0.12	1.84	0.57	1.73	0.24
17:1n7	0.25	0.23	0.29	0.18	ND	-
18:0	13.6	0.26	17.2	2.30	13.5	1.84
18:1n9***	25.9^a^	0.81	2.37^b^	0.17	25.0^a^	3.72
18:2n6t	ND	-	0.15	0.07	ND	-
18:2n6c	2.38	0.20	1.07	0.88	1.68	0.39
18:3n6	ND	-	0.25	0.02	ND	-
18:3n3	0.40	0.04	0.22	0.11	ND	-
18:2;9c11t	1.25	0.12	0.82	0.16	1.01	0.12
18:2;10t12c	0.44	0.13	ND	-	ND	-
18:2;10c12c	0.31	0.04	ND	-	ND	-
18:2;tt	0.37	0.05	ND	-	ND	-
20:0	ND	-	0.12	0.02	ND	-
20:1n9	ND	-	ND	-	ND	-
20:2n6	ND	-	0.32	0.01	0.27	0.25
20:3n6	ND	-	0.14	0.13	ND	-
20:4n6	ND	-	ND	-	ND	-
20:3n3	ND	-	ND	-	ND	-
20:5n3	ND	-	ND	-	ND	-
21:0	ND	-	ND	-	ND	-
22:0	ND	-	0.35	0.37	ND	-
22:1n9	ND	-	ND	0.00	ND	-
22:2n6	ND	-	0.28	0.48	1.59	0.32
22:6n3	ND	-	ND	-	ND	-
23:0	ND	-	ND	-	ND	-
24:0*	ND	-	6.04^b^	0.89	13.1^a^	1.56
24:1n9	ND	-	ND	-	ND	-
**Sums of fatty acids**						
SFA***	64.1^b^	0.69	88.6^a^	1.51	67.2^b^	3.51
MUFA***	30.8^a^	0.73	8.16^b^	0.81	28.2^a^	3.04
PUFA	5.14	0.17	3.27	1.10	4.54	0.58
n–3**	0.39^a^	0.04	0.15^b^	0.01	0.00^c^	0.00
n–6	3.56	0.11	2.23	1.05	3.25	0.03
**Ratio and indices**						
PUFA/SFA*	0.08^a^	0.00	0.06^b^	0.00	0.07^a^	0.01
AI***	2.18^b^	0.07	6.53^a^	0.75	1.94^b^	0.41
TI***	2.94^b^	0.07	9.35^a^	1.38	2.98^b^	0.43
HH**	0.74^a^	0.02	0.09^b^	0.03	0.82^a^	0.21
DA***	0.39^a^	0.00	0.15^b^	0.00	0.40^a^	0.05

ND: not detected; SD: standard deviation; SFA- saturated fatty acid; MUFA- monounsaturated fatty acid; PUFA- polyunsaturated fatty acid; AI- index of atherogenicity; TI- index of thrombogenicity; DA- desaturase activity; HH- hypocholesterolemic fatty acids/hipercholesterolemic fatty acids ^a,b,c^Means within the same row with different superscripts are significantly different. ANOVA (p<0.05) with Tukey´s post-hoc test *p<0.05

**p<0.01

***p<0.001.

### Desaturase activity (DA)

Fatty acid desaturases are enzymes that introduce double bonds into fatty acyl chains, thereby producing unsaturated and polyunsaturated fatty acids [36]. The Δ^9^ desaturase enzyme introduces double bonds at the Δ^9^ position of fatty acids [37].

The Δ^9^ desaturase activity was calculated using the ratio between fatty acids that are products and substrates for Δ^9^ desaturase. It was calculated as proposed by Lock and Garnsworthy [38]:

$$\text{DA}=(\text{sum of}\;\Delta^{9}\;\text{desaturase products})\;/\;(\text{sum of}\;\Delta^{9}\;\text{desaturase substrates}+\text{products})$$

### Statistical Analysis

All analyses were performed in triplicate and results were expressed as mean ± standard deviation (SD). A one-way analysis of variance was used to compare data obtained for different fermentation processes. When a significant *F* was found (*p* < 0.05), differences between means were evaluated by Tukey´s multiple comparison test using a commercially available statistical package (Systat software, Chicago, IL, USA). Two-side *p* values < 0.05 were considered statistically significant.

## Results and Discussion

### Chemical characteristics of fermented semi-skim milk with kefir grains

Table 1 reports the chemical characteristics variation during fermentation with different kefir grains. AD, AR and AV showed, as mean values of pH, 4.05±0.05, 4.08±0.01 and 4.00±0.05, respectively. This study followed kefir fermentation parameters in industrial scale (25°C for 18–24h) [39]. Thus, the pH values found in the present study are according to Ӧzdestan & Üren [39] which showed pH values of industrially produced kefir samples ranged from 4.11 to 4.53. Thus, all grains exhibited lower pH values than initial pH value of unfermented milk (*p* < 0.05). Titratable acidity averaged 105.22±1.70°D, 102.41±1.68°D and 104.31±1.68°D for AD, AR and AV respectively. The titratable acidity significantly increased after fermentation compared to unfermented milk. This increase confirms that lactose in milk was metabolized by kefir grains into lactic acid [17].

AD, AV and AR reached protein mean values of 5.94±0.04%, 5.94±0.11% and 5.98±0.23%, respectively (Table 1). These values were higher than protein content in unfermented milk (*p* < 0.05). Low pH has been reported as a sublethal stress factor for *Lactobacillus*, although it is widely used as a starter in the dairy industry for its ability to tolerate acidic conditions [14]. There is an increased level of gene expression of yeast cells in response to stress [40]. Moreover, the interaction between stress response proteins and lipid membrane unsaturation in bacterial cells has been described [41]. This interaction may explain the increase in protein level during fermentation in the present study. Additionally, there was no significant difference between the percentage of fat in fermented (with kefir grains) and unfermented milk (Table 1). Similarly, Yadav et al. [17] did not observe any change in total fat content in dahi, which is fermented milk-based food. Furthermore, there were not significant differences among kefir grains with respect to chemical characteristics.

**Fig 1:**
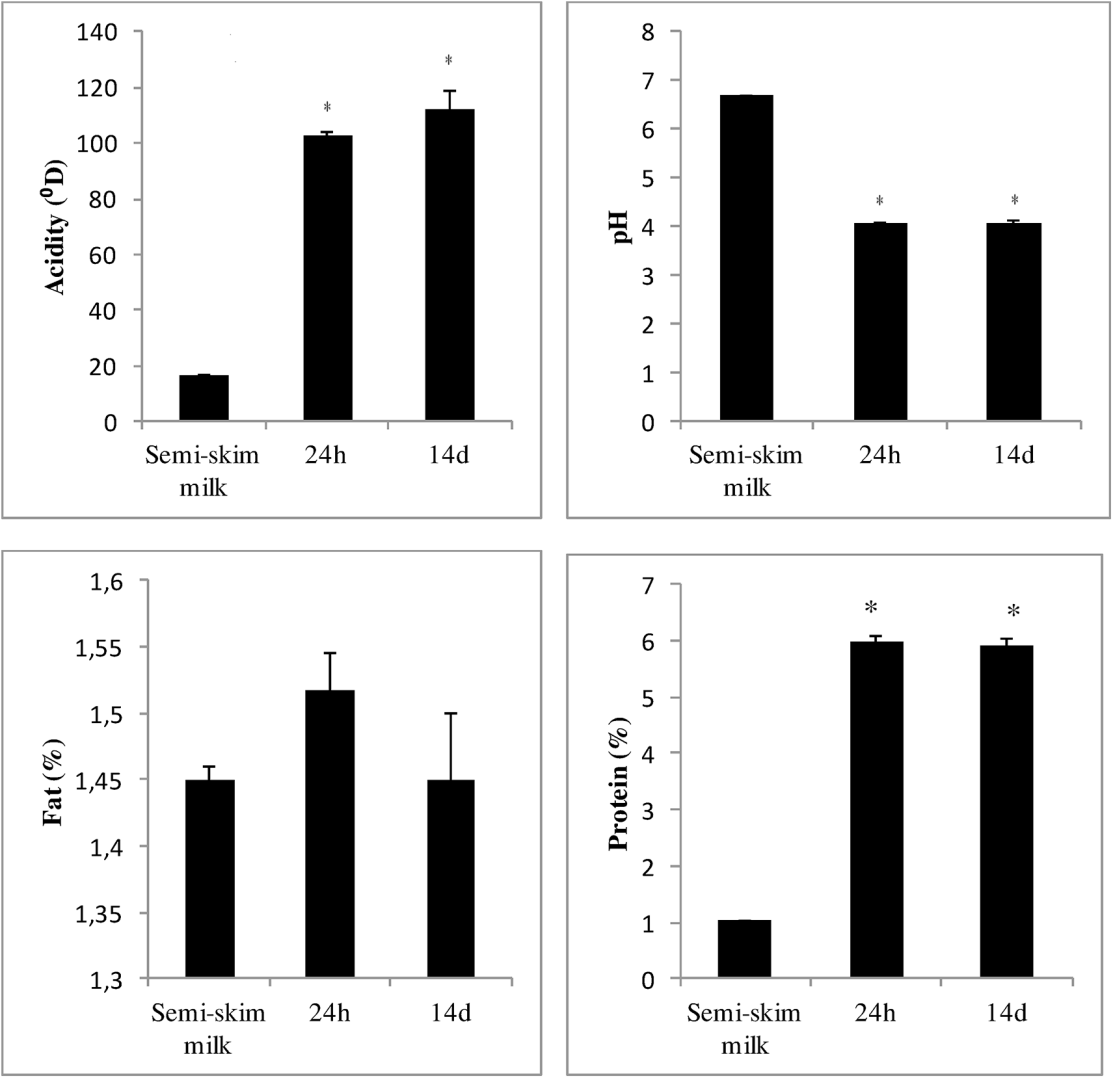
Chemical characteristics of fermented semi-skim milk with different storage time (0 and 14 days). Semi-skim milk was fermented with AR grain for 24h and after stocked for 14 days. Assays were performed in triplicate and the values reported are mean ± SD. (*) Significant difference among semi-skim milk, fermented milk for 24 h and storage for 14 days (p<0.05).

Fig 1 shows chemical characteristics during storage for AR grain. It is known that there are decrease in viable count and acid producing activity of lactic acid bacteria during storage, regardless of packaging or storage temperatures [42]. Pourahmad et al. [43] showed that the population of *Lactococcus* and *Lactobacillus* decreases during storage. Thus, no significant changes in chemical characteristics according to storage in the present paper could be explained by loss of cell viability during this storage period.

### Influence of kefir grains from different origins on the fatty acid and CLA content in fermented semi-skim milk

The composition of the fatty acids (g/100g total fatty acids) in semi-skim milk fermented by kefir grains is shown in Table 2. Oleic acid (18:1n9) content averaged 2.37, 3.98 and 24.7g/100g in AR, AD and AV, respectively. Thus, AR and AD grains had a significantly decreased oleic acid (18:1n9) amount when compared to unfermented milk (25.9g/100g). The consumption of oleic acid (18:1n9) in this study can be attributed to the fact that unsaturated fatty acids can be converted to products which increase membrane rigidity and decrease the permeability of the membrane to H^+^, Na^+^ and possibly to H_2_O_2_ during stress conditions including fermentation [14].

Mean values of tetracosanoic acid (24:0) were 6.04 and 15.6g/100g in AR and AD respectively, and this fatty acid was not detected in AV. Thus, AR and AD had tetracosanoic acid (24:0) values significantly increased during fermentation. This may be explained because stress factor was reported to induce changes in fatty acids according to the degree of fatty acid unsaturation, cyclization and proportions of long-chain fatty acids containing 20 to 24 carbons in *Lactobacillus* [44]. These factors (consumption of oleic acid and production of tetracosanoic acid) may have contributed to increasing saturated fatty acids in fermented milk with grains (AD and AR) when compared to semi-skim milk (*p* < 0.05).

Palmitic acid (16:0) content found in AD, AR and AV was 24.5, 29.4 and 36.6g/100g. Only AD grain significantly decreased palmitic acid (16:0) during fermentation while AV grain had significant increase of this fatty acid with respect to unfermented milk (29.9g/100g) (Table 1). Epidemiological [45] and *in vivo* studies [46] demonstrate that consumption of fermented milk can reduce the incidence of cancer. Palmitic acid (16:0) has been shown to be an antimutagenic component present in milk fat, the concentrations of which are greater in kefir than in milk and yogurt. The acetone-extracted kefir, which is rich in fat portions, showed higher antimutagenic potential against several chemical mutagens in Ames Salmonella microsomal test when compared to extracted milk and yogurt [16]. Furthermore, it was showed that palmitic acid is able to inhibit mutagenesis in dose-dependent response in bacterial cells *in vitro* [7]. Regarding ingested fatty acids, it is known that local tissue concentrations easily reach levels 10-fold greater than plasma concentrations [47]. Therefore AV may have been able to increase antimutagenic potential in fermented milk. Thus, in general, the fatty acids composition was significantly different among the different kefir grains.

Guzel-Seydim et al. [16] showed that milk fermented with kefir had higher contents of oleic, palmitoleic, vaccinic, eicosaneoic, erucic and linoleic acids when compared to unfermented milk. The difference between the Guzel-Seydim et al study and the present study regarding the fatty acid content between different grains may be due to the disparate microbial communities of kefir grains [25]. Each bacterial group shows different fatty acids production potentials [48]. Bacteria use the disassociated fatty acid synthesis II pathway, thus multiple discrete enzymes synthesize the fatty acid chain. Fatty acids are assembled two carbons at a time through of condensations cycles [49]. The use of different bacterial cultures had significant effect on fatty acid composition of white pickle cheese [21].

Nutritional quality indexes and ratios of milk fermented with different kefir grains are showed in Table 2. SFA content was 72.7, 84.8 and 88.6g/100g for AV, AD and AR, respectively. The amounts of MUFA found were 7.23, 8.16 and 25.8g/100g for AD, AR and AV, respectively. Therefore, there was significant increase of SFA and MUFA reduction after fermentation for all grains, regardless of the origin of the kefir grains, with respect to unfermented milk (64.1 and 30.8g/100g for SFA and MUFA, respectively). AI mean values were found to be 3.25, 3.33 and 6.53 for AV, AD and AR, respectively. Despite the increase of SFA and reduction of MUFA during fermentation, there was no significant difference in AI as compared to unfermented milk, which is AI = 2.18, except for the AR grain which showed a significantly elevated AI (6.53). This is in accordance with Stajić et al. [50], which related AI increase in fermented products. Furthermore, Nantapo et al. [15] found AI values in cow milk (4.08–5.13) close to the value of milk fermented with AR grain at the present study (6.53). AI in semi-skim milk (2.18) is in accordance with what was found by Hernández et al. [51] for milk, ranging from 2.12 to 2.27. On the other hand, TI values averaged 4.57, 9.35 and 9.36 for AV, AR and AD, respectively. Thus, trombogenicity indexes were significantly higher to AR and AD compared to unfermented milk (2.94). It may be that TI also increases with fermentation, just as it had for AI; however, no data about TI in fermented product has been found in the literature. The increase of AI and TI during fermentation may be related to the starter culture used to ferment food. TI values in semi-skim milk at this study (2.94) are in accordance with the ones found by Hernández et al. [51], which ranged from 2.51 to 2.99 (Table 2).

AV, AR and AD showed as mean values of PUFA/SFA ratio 0.02, 0.04 and 0.09, respectively. Meanwhile, PUFA values found for AV, AR and AD were 1.50, 3.27 and 7.99, respectively. As a consequence of SFA increase, AV and AR grains reduced PUFA/SFA ratio after fermentation compared to unfermented milk (0.08). As AD showed higher PUFA content than other grains, the PUFA/SFA ratio was higher in AD when compared to other grains. Thus, no grain or even milk before fermentation had PUFA/SFA ratio above 0.4 as recommended by health guideline, and therefore it may have detrimental effect on LDL cholesterol plasmatic level [33]. This can be explained by the fact that milk is a product relatively rich in saturated fatty acids and low in unsaturated fatty acids. Results in this study in accordance with Caldeira et al [52] that found PUFA/SFA values ranged between 0.03–0.05 in buffalo milk. PUFA/SFA reduction in AV and AR during fermentation in this study is aligned with Stajić et al. [50] who found PUFA/SFA values reduced during fermentation. HH values in this study were 0.09, 0.25 and 0.53 for AR, AD and AV, respectively. The HH value was reduced in AR and AD grains with respect to unfermented milk (0.74) (*p*<0.05). Thus, the fermentation can compromise the nutritive value of milk because it can lead to increase of hypercholesterolemic fatty acids in relation to hipocholesterolemic fatty acids. The HH value in present study is according to Osmari et al. [53] who found an HH mean value of 0.75 in goat milk. No HH value in literature for fermented products was found.

Table 2 reports Δ9-desaturase activity in kefir grains of different origins. AR, AD and AV showed as mean values of Δ9-desaturase activity 0.12, 0.15 and 0.31, respectively. Thus, Δ9-desaturase activity was significantly higher in AV compared to AR and AD grains, which may have contributed to higher resistance for AV in fermentation conditions, since it is known that there is activation of fatty acids desaturation pathway in stress conditions in order to protect the cell from damage [14]. Additionally, this greater desaturase activity in AV may also have contributed to decrease SFA and increase MUFA in relation to AD and AR grains (*p*<0.05) (Table 2). Saturated fatty acids were most abundant in AD and AR (84.8 and 88.6g/100g, respectively). Although this level is relatively high compared with AV grain (72.7g/100g) it is in accordance with Serafeimidou et al. [19] that found SFA yields varying from 72.98 to 90.11 (g/100g total fatty acids) in commercial fermented cow milks. AV showed higher concentration of MUFA (25.8g/100g) in relation to other grains (p<0.05) which is slightly lower than reported by Florence et al. [54] who found MUFA amounts varied from 27.1 to 27.3% in fermented cow milks. There no was significant difference in PUFA content between kefir grains. Furthermore, unfermented milk in this study had a similar content of SFA and MUFA (64.1 and 30.8g/100g), respectively (Table 2), when compared to that found by Florence et al. [55], which ranged from 64.5% to 70.4% SFA and 25.9% to 31.8% MUFA. However, the PUFA content was slightly higher in this study (5.14g/100g). Stearic acid (18:0) and tetracosanoic acid (24:0) increased during fermentation in AD (*p*<0.05); though there was an increase of lauric acid (12:0), pentadecanoic acid (15:0) and also tetracosanoic acid (24:0) in AR (*p*<0.05) (Table 2). This fact may have contributed to SFA higher in AD and AR. The lack of statistically-significant consumption of oleic acid (18:1n9) and of production of tetracosanoic acid (24:0) in AV may be due to a possible resistance and, consequently, greater viability of AV relatively to other grains during fermentation conditions, which may have contributed to lower SFA amounts and higher MUFA amounts in this grain (Table 2). Therefore, AD, AR and AV were able to significantly change the fatty acids composition compared to unfermented milk.

AD was the single grain able to produce CLA in semi-skim milk (Table 2). The *cis9*,*trans11* isomer was produced in higher concentration (72.2% of total CLA), while *trans10*,*cis12* and *cis10*,*cis12* contributed to 13.2% and 14.6%, respectively, of total CLA. The 18:2,*tt* isomer was not produced by AD (Table 2). However, there was no significant production of CLA by different kefir grains during fermentation. *Lactobacillus acidophilus* and *Lactobacillus casei* use linoleic acid produced by lipolysis of milk fat as a substrate for CLA synthesis, which is then converted to stearic acid (C18:0) [17]. In the present study, AD had a significantly higher stearic acid (18:0) level compared to the other analyzed grains. This observation may be due to higher lipase activity in AD, which confirms the fact that AD was the only grain able to produce CLA. In addition, the time fermentation of 24h could have been enough long to lead to complete conversion of CLA to stearic acid (18:0) [23].

### Influence of storage time on fatty acid and CLA content in fermented semi-skim milk

The Table 3 presents the fatty acids content (g/100g total fatty acids) in AR grain analyzed after 24h of fermentation and 14 days of storage. AR showed oleic (18:1n9) acid content of 2.37 and 25.0g/100g after fermentation and storage, respectively, while mean values of tetracosanoic acid (24:0) were 6.04 and 13.1g/100g after fermentation and storage, respectively. Thus, these fatty acids presented significantly higher amounts during storage. On the other hand, lauric acid (12:0) content presented by AR was 7.45 and 1.88g/100g after fermentation and storage, respectively. Furthermore, pentadecanoic acid (15:0) amount found was 24.4 and 3.1g/100g after fermentation and storage, respectively. Lauric (12:0) and pentadecanoic (15:0) acids significantly decreased during storage. The reduction of pentadecanoic acid (15:0) could be related to an oxygen-consuming desaturase system for acid lactic bacteria, with a consequential increase in fatty acid desaturation as a cellular response to environmental stress. The unsaturation level is considered the most important response to the various combinations of sublethal stresses applied to the *Lactobacillus* strain [14]. Moreover, studies have shown that linoleic acid (18:2n6) biosynthesis in *Escherichia coli* provides survival advantages in the stationary phase [56]. Δ9-desaturase activity was measured as 0.15 and 0.40 after fermentation and storage, respectively. In fact, there is significant increase of Δ9 desaturase activity during storage compared to fermentation (Table 3). Thus, Δ9 desaturation system with consequent production of oleic acid can be more important during stress conditions due to storage than those due to fermentation conditions. SFA content averaged 88.6 and 67.2g/100g after fermentation and storage, while MUFA content averaged 8.16 and 28.2g/100g after fermentation and storage, respectively (Table 3). Temperature, atmosphere, exposure to light, and relative humidity affect the stability of bacteria during storage. These factors alone or in combination might lead to a high loss of viable cells and/or acid-producing capacity due to deteriorating chemical reactions [57]. The stress due to storage could lead to a decrease in saturated fatty acids and an increase of long-chain fatty acids biosynthesis by lactic acid bacteria in order to reach appropriate proportions of fatty acid unsaturation and fatty acid chain length in cellular membrane and thus to alter fatty acid composition. This would be consistent with the results in the present study, since there is significant increase of desaturase activity during storage relatively to fermentation, and also decrease of SFA (67.2g/100g) and significant increase MUFA (28.2g/100g) during storage (Table 3). These findings are similar to Florence et al. [54] that established SFA content after storage of fermented cow milk slightly higher (68.5% to 69.2%), whereas the MUFA level presented slightly lower levels (27.9% to 28.1%) than those found in present study. With respect to nutritional quality indexes and ratios, AI values found were 6.53 and 1.94g/100g after fermentation and storage, respectively; TI values were measured as 9.35 and 2.98g/100g after fermentation and storage, respectively; HH was showed as being 0.09 and 0.82 after fermentation and storage, respectively and PUFA/SFA ratio was established as 0.06 and 0.07 after fermentation and storage, respectively. As a consequence of SFA decrease and an increase of unsaturated fatty acids, AI and TI values decreased during storage compared to fermentation (*p*<0.05) (Table 3), whereas PUFA/SFA ratio and HH index increased with storage (*p*<0.05). Thus, storage can improve the nutritional value of fatty acids content when compared to fermentation. No PUFA/SFA value and HH index for fermented storage product was found in the literature.

The molecular complex of bovine milk protein and oleic acid (18:1n9) is able to kill human cancer cells *in vitro* and inhibits tumor growth in a rat bladder tumor model, although both cases demonstrate dose-dependent sensitivity [5]. This molecular complex killed all the human urothelial cell cancer cell lines tested in dose-dependent sensitivity ranging from 0.15 to 0.8mg/mL. Thus, the high oleic acid (18:1n9) amount reached in storaged milk (25.0g/100g) may make it to become potentially antimutagenic and anticarcinogenic.

There was no significant difference in stearic acid (18:0) between fermented milk and storage fermented milk, indicating that there was no significant change in lipase activity during storage, which may be explained by the decreased cellular viability of acid lactic bacteria. There was no significant difference in CLA amount between fermentation and storage (Table 3) in the present study. This fact also can be explained by decrease of cellular viability during storage, which would decrease CLA production ability. The result is in agreement with Yadav et al. [17] who showed that CLA content remained constant until day 10 of storage.

### Influence of dairy matrix on fatty acid and CLA content

The fatty acids composition (g/100g total fatty acids) of the AD grain analyzed after 24h of fermentation of both semi-skim milk and whole milk is presented in Table 4.

**Table 4:** Comparison of fatty acid composition (g/100g total fatty acids) in semi-skim milk and whole milk both fermented with AD kefir grain for 24h.

Fatty acids	Semi-skim milk		AD		Whole milk		AD	
	Mean	SD	Mean	SD	Mean	SD	Mean	SD
6:0	1.17	0.26	ND	-	ND	-	ND	-
8:0	1.44	0.43	ND	-	0.57	0.00	ND	-
10:0**	5.01^a^	0.06	1.28^b^	1.52	2.08^ab^	0.50	0.41^b^	0.21
12:0	2.65	0.22	2.06	2.10	2.75	0.63	0.84	0.20
13:0	ND	-	0.24	0.05	0.14	0.03	0.22	0.04
14:0**	10.8^a^	0.68	8.84^a^	6.28	11.1^a^	1.59	5.20^b^	0.24
14:1n5	2.21	0.37	1.77	1.23	1.15	0.17	1.05	0.07
15:0	1.29	0.08	0.67	0.00	1.27	0.12	0.67	0.00
15:1n5	0.31	0.01	0.20	0.20	0.34	0.03	0.10	0.14
16:0***	29.9^b^	0.87	24.5^c^	0.22	34.2^a^	0.81	24.5^c^	0.13
16:1n7	2.10	0.12	2.30	0.58	1.83	0.08	1.96	0.07
17:1n7	0.25	0.23	ND	-	0.34	0.02	ND	-
18:0***	13.6^b^	0.26	39.7^a^	2.77	13.9^b^	1.41	38.0^a^	0.62
18:1n9***	25.9^a^	0.80	3.98^b^	0.03	26.4^a^	2.23	3.97^b^	0.01
18:2n6t	ND	-	0.15	0.14	ND	-	0.08	0.12
18:2n6c	2.38	0.20	2.92	0.39	2.08	0.17	2.68	0.04
18:3n6	ND	-	0.57	0.07	0.22	0.08	0.60	0.05
18:3n3	0.40	0.04	0.28	0.05	0.38	0.02	0.26	0.02
18:2;9c11t	1.25	0.12	1.12	0.18	1.09	0.07	1.22	0.04
18:2;10t12c	0.44	0.13	0.19	0.17	ND	-	0.28	0.07
18:2;10c12c	0.31	0.04	0.23	0.05	ND	-	0.25	0.01
18:2;tt	0.37	0.05	ND	-	ND	-	ND	-
20:0	ND	-	0.26	0.06	0.24	0.05	0.26	0.09
20:1n9	ND	-	0.13	0.12	0.11	0.10	ND	-
20:2n6	ND	-	0.50	0.28	ND	-	0.33	0.03
20:3n6	ND	-	0.32	0.07	ND	-	0.36	0.03
20:4n6	ND	-	ND	-	ND	-	ND	-
20:3n3	ND	-	ND	-	ND	-	ND	-
20:5n3	ND	-	ND	-	ND	-	ND	-
21:0	ND	-	ND	-	ND	-	ND	-
22:0	ND	-	ND	-	ND	-	ND	-
22:1n9	ND	-	0.18	0.16	ND	-	0.27	0.01
22:2n6	ND	-	1.70	0.45	ND	-	1.44	0.07
22:6n3	ND	-	ND	-	ND	-	ND	-
23:0	ND	-	ND	-	ND	-	ND	-
24:0***	ND	-	15.6	1.80	ND	-	14.5	0.74
24:1n9	ND	-	ND	-	ND	-	ND	-
**Sums of fatty acids**								
SFA***	64.1^b^	0.69	84.8^a^	0.37	66.0^b^	2.17	84.7^a^	0.78
MUFA***	30.8^a^	0.73	7.23^b^	0.60	30.2^a^	1.97	7.55^b^	0.15
PUFA**	5.14^ab^	0.17	7.99^a^	0.80	3.76^b^	0.20	7.52^a^	0.25
n–3*	0.39^a^	0.04	0.28^bc^	0.05	0.37^ab^	0.02	0.25^c^	0.02
n–6*	3.56^bc^	0.11	6.76^a^	1.21	2.34^c^	0.18	6.04^ab^	0.19
**Ratios**								
PUFA/SFA*	0.08^ab^	0.00	0.09^a^	0.01	0.06^b^	0.00	0.09^ab^	0.00
AI	2.18	0.07	3.33	0.16	2.49	0.39	3.33	0.16
TI***	2.94^b^	0.07	9.36^a^	0.81	3.39^b^	0.26	8.91^a^	0.21
HH*	0.74^a^	0.02	0.25^b^	0.01	0.68^a^	0.08	0.25^b^	0.01
DA***	0.39^a^	0.00	0.15^b^	0.00	0.36^a^	0.02	0.15^b^	0.00

ND: not detected; SD: standard deviation; SFA- saturated fatty acid; MUFA- monounsaturated fatty acid; PUFA- polyunsaturated fatty acid; AI- index of atherogenicity; TI- index of thrombogenicity; DA- desaturase activity; HH- hypocholesterolemic fatty acids/hipercholesterolemic fatty acids ^a,b,c^Means within the same row with different superscripts are significantly different. ANOVA (p<0.05) with Tukey´s post-hoc test *p<0.05

**p<0.01

***p<0.001.

Myristic acid (14:0) ranged from 10.8 to 8.84g/100g, PUFA level ranged from 5.14 to 7.99g/100g and DA ranged from 0.39 to 0.15 after fermentation in semi-skim milk, while in whole milk myristic acid (14:0) ranged from 11.1 to 5.20, PUFA from 3.76 to 7.52g/100g and DA from 0.36 to 0.15 after fermentation. Thus, there is a significant decrease of myristic acid (14:0) and a significant increase of PUFA after fermentation in whole milk only. This desaturation trend found in this study for high-lipidic matrix is in accordance to literature. Milk and dairy products also represent a source of lipids and cholesterol [58]. Several studies have suggested cholesterol removal capability of lactic acid bacteria [59, 60]. Furthermore, assimilation of cholesterol in milk by kefir cultures was reported [61]. The cholesterol incorporation into the cellular membrane alters the fatty acid contents and also increases desaturation of fatty acids to maintain membrane fluidity [18]. In addition, Piironen et al. [62] observed cholesterol content correlated with fat content in milk products, which would lead to a higher desaturation level in higher lipid content dairy products. Thus, Serafeimidou et al. [19] observed the lowest amounts of saturated fatty acids and palmitic acid (16:0) in high-lipid yogurts. However, no significant difference in Δ9-desaturase activity between both fermented matrices (0.15) (Table 4) has been observed in this study. Thus, the PUFA increase during fermentation only in whole milk could be explained by highest desaturase activity of others types in fermented whole milk.

Palmitic acid (16:0), oleic acid (18:1n9) and MUFA amounts decreased after fermentation, while stearic acid (18:0), tetracosanoic acid (24:0) and SFA amounts increased after fermentation regardless of dairy matrix (*p*<0.05). After fermentation both in semi-skim milk and whole milk, they had high tetracosanoic acid (24:0) concentrations (*p*<0.05), which is proportional to the increase of long-chain fatty acids containing 20 to 24 carbons in stress conditions during fermentation [44]. The increase of stearic acid (18:0) can be related to lipase activity and the conversion of linoleic acid to stearic acid. High concentrations of tetracosanoic (24:0) and stearic (18:0) acids can have contributed to increasing saturated fatty acids in milks after fermentation (Table 4).

TI mean values ranged from 2.94 to 9.36 after fermentation in semi-skim milk and from 3.39 to 8.91 in whole milk after fermentation. Thus, there was a significant increase in TI and no significant difference between AI and PUFA/SFA ratio after fermentation in both matrices (*p*<0.05). There was the reduction of HH after fermentation in both fermented milks to 0.25 (*p*<0.05). Thus, the HH index was not influenced by the dairy matrix.

## Conclusions

The origin of Kefir grains influences the change of the fatty acid contents in semi-skim milk. AD and AR grains are the most capable of changing the fatty acid contents. AV increased the palmitic acid content in milk, thus it may have been able to increase antimutagenic potential in fermented milk. The storage time leads to changes in fatty acid compositions in milk, so that stocked milk can have higher anticarcinogenic potential due to the high amount of oleic acid. Furthermore, storage can be used to decrease SFA and increase MUFA levels. Only high-lipidic matrix showed an increase of PUFA after fermentation. The CLA concentration did not increase significantly among kefir grains, storage time and fat concentration of the milk. Total fat was the only chemical characteristic not altered during fermentation and storage. Acidity and protein content increased while pH decreased during fermentation, but not due to storage time. Additionally, studies intending to optimize desaturase activity during fermentation conditions may contribute to obtaining a fermented product with a higher nutritional lipid quality.
